# Clinical Prediction of Suicide and Undetermined Death: A Pseudo-Prospective Clinical and Medico-Legal Study of Substance Abusers

**DOI:** 10.3390/ijerph14030310

**Published:** 2017-03-17

**Authors:** Louise Brådvik, Mats Berglund, Arne Frank, Peter Löwenhielm

**Affiliations:** 1 Department of Clinical Sciences, Division of Psychiatry, Lund University, SE-221 85 Lund, Sweden; mats.berglund@med.lu.se; 2 Forensic Medicine, Lund University, SE-221 85 Lund, Sweden; peter.lowenhielm@sjobo.nu

**Keywords:** substance use disorders, death of undetermined intent, suicide, suicide attempt, suicidal ideation, prediction, autopsy findings

## Abstract

This study examines aspects of prediction of suicide and death of undetermined intent. We investigated all consecutive, autopsied patients between 1993 and 1997 who had been in contact with the Addiction Centre in Malmö from 1968 onwards. The staff was asked, shortly after autopsy but before they knew of the manner of death, if they thought the patient had committed suicide. The case records were blindly evaluated, and toxicological autopsy findings for alcohol in blood samples investigated. The specificity of prediction was 83% and significantly more often correct than the sensitivity, which was only 45% for suicide and for suicide/death of undetermined intent (93% versus 39%). Suicidal communication was more often considered non-serious before death of undetermined intent than before suicide. The former could be predicted by ideation but not by suicide attempt reported in case records, unlike suicide, which was predicted by both. The undetermined group also showed higher levels of alcohol in the blood at autopsy. We concluded that more serious clinical investigation of suicidal feelings, which may be hidden and not taken seriously, and treatment of alcohol use disorders with active follow-up appear urgent in the efforts to prevent suicide.

## 1. Introduction

Suicide is a major health problem, with more than 800,000 people killing themselves every year [1]. The prediction of accomplished suicide in order to prevent it is urgent but difficult. Different rating scales have been developed, such as the Scale of Suicidal Ideation (SSI) [2], the Suicidal Intent Scale (SIS) [3], and the Sad Persons Scale [4]. However, a recent review of the predictive value for future suicide attempts has not been encouraging [5], with the conclusion that none of the known rating scales reached a sensitivity of 80%, nor was a specificity of 50% reached. Clinical judgement as a complement to rating scales was strongly recommended. Typical scales for suicide prediction can be less effective when a respondent has alcohol or other substance use disorders, so additional clinical judgement is warranted.

Alcohol use disorders are also commonly found among suicide victims [6,7,8]. These are usually regarded as the second most common diagnosis among suicide victims after depression, and have been related to deaths of undetermined intent [8,9,10]. In population-based surveys, suicide and death of undetermined intent are usually combined in the analyses [11,12], and similarities have been shown between the two groups in several studies [13,14,15]. However, although there are similarities between these manners of death, differences in background variables have been highlighted recently [10], as well as similarities between accidental overdoses and death with undetermined intent rather than with suicide in substance use disorders [16].

Alcohol and other substance use disorders also show a high risk of suicide [17,18]. Suicidal ideation and suicide attempts are also common among people with alcohol use disorders [19]. However, predictors such as suicide attempts and suicidal ideation are often considered less serious among people with alcohol use disorders, especially if occurring during drinking and intoxication. Though attempted suicide has also been shown to be a highly significant risk factor for completed suicide in young males with alcohol use disorders, this group was found to have a significantly lower risk of completed suicide than other suicide attempters [20]. Likewise, the suicide risk in suicide attempters who had recently consumed alcohol was assessed as less severe, and they were less often referred to a psychiatrist compared with those who had not [21]. Other investigators have found that a correlation between suicidal intent and the lethality of the suicide attempt was seen only among patients without a diagnosis of alcohol dependence [22]. Low scores on the SIS when alcohol was used prior to self-harm have also been shown [23].

However, other authors have pointed out the triggering effect of alcohol on suicidal behaviour. According to one study, 50% of attempted suicides happened within one hour of alcohol use [24]. Attempted suicide can be triggered by alcohol [25], and alcohol use has been associated with a faster transition from suicidal impulse to action [26]. High levels of alcohol at autopsy have been found in people with brittle/sensitive personalities [27], and a dose-response relationship between the number of drinks and suicidal behaviour has also been shown [28].

The present study is based on a sample of patients who had been treated at the Addiction Centre in Malmö from 1968 onwards, who died in the period from 1993 to 1997, and who were autopsied at the Department of Forensics. The sample has been presented in a previous study on unnatural death and drugs used in life [29]. This special sample is now revisited, with the study inspired by recent findings and discussions of the limited sensitivity and specificity of known rating scales for suicide risk. Complementary clinical evaluation was recommended.

The first aim was to investigate the accuracy of the staff’s clinical prediction of suicide and death of undetermined intent, and possible differences between these manners of death. A second aim was to investigate, using case record evaluation, the predictive value of suicidal ideation and attempt of suicide and death by undetermined intent. Finally, alcohol levels at autopsy by those manners of death were compared.

## 2. Material and Methods

A forensic examination sampling procedure was used. The procedure was carried out on all consecutive autopsies of patients who had been in contact with the Addiction Centre in Malmö University Hospital. In Sweden, a forensic examination is carried out on most people who have died outside hospitals by suspected natural causes (disease) but with no medical history that can explain the death, or by unnatural manner (trauma including homicide, suicide, death of undetermined intent, and accidental fatal intoxications).

**Figure 1:**
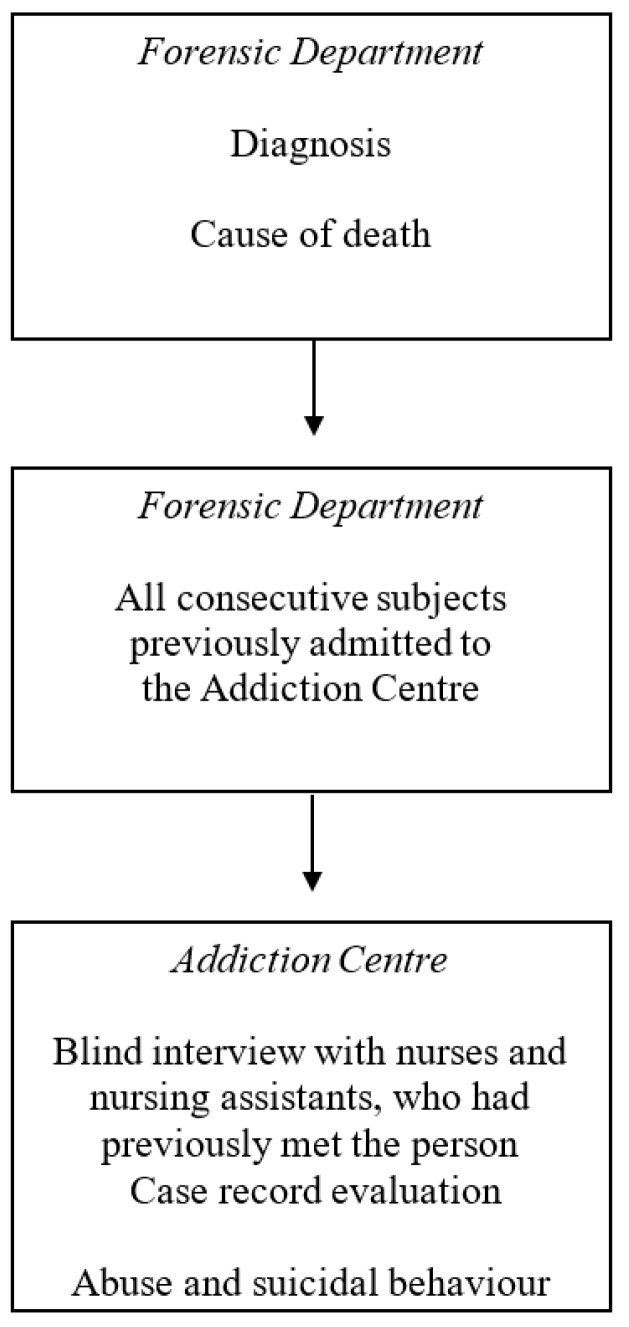
Sampling procedure (modified after [29]).

In all, 388 consecutive forensic autopsies on previous patients at the Department of Forensic Medicine in Lund from 1993 to 1997 inclusive were investigated (Figure 1), as well as an investigation of case records from 1968 onwards. The sampling was carried out in the 1990s, but there have been no significant changes in methodology since then, and changes in the epidemiology of deliberate self-harm do not apply to suicidal ideation and attempt investigated in the present sample.

Substance use was diagnosed according to International Classification of Disease (ICD 9 and 10) [30,31] for all inpatients, who constituted 76% of the sample. The remaining 24% had been admitted as outpatients, and had applied because they subjectively considered themselves to have a substance use problem. It is safe to conclude that they all fulfilled the criteria for alcohol dependence and/or had a drug problem. From 1968 to 1994, all the patients treated at the Department of Clinical Alcohol Research were admitted for alcohol problems; after that date, the clinic became an Addiction Centre, which also received patients with narcotic misuse. Some of them may not have had an alcohol problem, but in only seven cases could alcohol use disorder not be confirmed (1.8% of the total sample), though it could be suspected. A total of 89 patients had used illegal drugs (some legal drugs as well) and another 73 had used legal drugs. A previous study had shown that the number of drugs used in life increased the risk of death of undetermined intent but not suicide [29]. However, number of drugs detected at autopsy showed similar rates for undetermined intent and suicide.

The interviews were performed within a few days of the patient’s death, with nurses and nursing assistants who had previously had contact with the patient. Most of the services provided involved psychosocial interventions by nurses and registered nurses; the group of physicians was small and the younger physicians had often only worked at the department for a few months. We therefore decided to interview nurses/registered nurses to ensure that we acquired the most reliable information.

As the interviews were performed shortly after death, the interviewer and the interviewees did not know the manner of death. The staff remembered 157 patients, as expected more often those with a recent contact (Table 1). However, there was no difference in remembering manner of death. The suicidal outcome as judged by staff was dichotomized into ‘yes’ or ‘no’, but they sometimes stated that it was ‘only a threat’ or ‘just when drunk’, which made up a third category ‘intent not considered serious’.

The entire records were evaluated for those who had been in- or outpatients at the Addiction Centre in Malmö University Hospital from first admission and onwards. Consequently, these ratings were not biased by any knowledge of the manner of death. The ratings of suicidal ideation and attempt were used in the present study.

There were similar rates of patients who had been in contact with the clinic within the previous three months, regardless of manner of death. Furthermore, there was no evidence of more people seeking help in later contact in future suicide victims or those who died by undetermined intent (27% of the suicide cases, 30% of the undetermined cases, and 23% of the others, see Table 2. There were also similar numbers for other time intervals, one year, five years, etc. We chose to include all remembered patients, regardless of time passed since last contact, and then compared those with recent contact with more distant.

Femoral blood samples of alcohol concentration had been collected for in 43/45 cases of suicide and in 86/91 of deaths by undetermined intent.

### 2.1. Statistics

A logistic regression with odds ratio (OR) and confidence interval (CI) was used to relate suicidal ideation and attempt to undetermined death and completed suicide. Fisher’s exact test was used for comparison between groups. Student’s *t*-test was used for comparison of continuous variables.

### 2.2. Ethical Considerations

Ethical approval was not required for deceased persons in Sweden at that time. However, the National Board of Forensic Medicine approved the study by oral confirmation number 1993.

## 3. Results

### 3.1. Staff Judgement

Suicide predicted by staff and ‘intent not considered serious’ were compared for suicides and death of undetermined intent (Table 3). A significantly higher incidence of ‘intent not considered serious’ was found in the undetermined group (8/17 versus 0/9, Fisher’s exact test *p* = 0.023).

#### 3.1.1. Suicide versus Non-Suicide

Sensitivity was 9/20 (45%) for the prediction of future suicide, but specificity was high 114/137 (83%). The latter prediction was significantly more often correct as compared to the prediction of suicide (*p* < 0.0001).

#### 3.1.2. Suicide and Undetermined Intent versus Other Manners of Death

The sensitivity was 18/67 (26%) if suicide and death of undetermined intent were aggregated. If ‘intent not considered serious’ was included, the sensitivity was higher 26/67 (39%), more similar to suicide, and the specificity was also high, 84/90 (93%).

The prediction of no suicide/death of undetermined intent was significantly more often correct, as compared to the prediction for suicide/undetermined intent (84/90 versus 26/67, *p* < 0.0001).

The figures were similar for those with recent contact within a year and for the total group.

The prediction of neither suicide nor death of undetermined intent was 20/20 (100%), while the prediction of suicide/death of undetermined intent was 7/18 (39%) (*p* < 0.0001).

### 3.2. Suicidal Behaviour Reported in the Case Records Related to Suicide and Undetermined Death

The association between suicidal behaviour (ideation and attempt) reported in the case records and manner of death is presented in Table 4. The sensitivity of the prediction of suicide by suicide ideation was 36%, and the specificity was 84%. The sensitivity of prediction by suicide attempt was 33% and the specificity was 83%. The sensitivity of prediction of suicide/undetermined death by suicidal ideation was 29% and the specificity was 88%. The sensitivity of prediction of suicide/undetermined death by suicide attempt was 26% and the specificity was 83%. In all cases the sensitivity was lower than the specificity.

The sensitivity ranged from 26% (suicide attempt in case records for suicide/undetermined intent) to 45% (prediction by staff, regardless of time span since last contact.)

The specificity ranged from 83% (suicide attempt in case records for suicide) to 100% (prediction by staff within a year before suicide). Thus, a somewhat better prediction was made by staff.

A logistic regression was performed to assess the impact of suicidal ideation and suicide attempt on suicide and undetermined death. Suicidal ideation was related to both suicide (OR: 2.95, CI = 1.50–5.81, *p* = 0.002) and death of undetermined intent (OR: 1.92, CI = 1.09–3.37, *p* = 0.023). However, suicide attempt was only related to suicide (2.41, CI = 1.22–4.75, *p* = 0.011), and there was no significant correlation with death of undetermined intent.

### 3.3. Toxicological Autopsy Findings

The blood levels of alcohol were higher in death of undetermined intent (1.79 per mille) as compared to suicide (0.72 per mille) (*t*-test *p* = 0.0001).

In the suicide group, alcohol was detected in 25/43 (58%) versus 60/82 (73%) in the undetermined group, a non-significant difference. One person with a positive test in urine was included.

## 4. Discussion

The present study considers clinical prediction without the use of any rating scales. This prediction did not seem to be very accurate for suicide, less than 50% with or without the inclusion of death of undetermined intent. On the other hand, the specificity was high, and the prediction that persons would *not* commit suicide was often correct (83%–93%). When suicide attempt or ideation were used as predictors, a similar sensitivity and specificity showed the same relationship with a higher specificity.

However, the highest specificity was found by staff intuition within a year before death.

This contradicts the conclusion by the Swedish Council on Health Technology Assessment (SBU) review of questionnaires [5], which showed a low specificity and higher sensitivity, mostly including repeated suicide attempts rather than completed suicide. A meta-analysis of suicide risk within a year after discharge showed that 60% of future suicide victims were considered to be at low risk at discharge, so 40% were high risk, similar to the present finding (39%–45%) [32]. Prediction by staff intuition could be a complement to rating scales.

The poor prediction of suicide may reflect unawareness of life events occurring after last contact that may trigger suicide. The staff could judge resilience only, which corresponds to trait factors in suicide risk but not state as described by Goldston et al. [33]. Better follow-up may improve the possibility of providing support in the event of distressing life events.

The better prediction of survival may also reflect better contact. The staff seemed to know who would survive, but they did not know who would commit suicide, and prediction had escaped their attention. Ringel [34] proposed a presuicidal syndrome, which included ‘Einengung’ or constriction of human relationships and values. In this state, the person may be very much alone and not communicative with others. Furthermore, in an investigation of the long-term course of depression after a suicide attempt [35], some subjects pointed out that the decision to continue living was a very private one, not necessarily communicated with others. Therefore, the decision to commit suicide may very well be a decision taken in a lonely state.

The present findings support both views, which may be an explanation for the poor sensitivity and a need to be more open to exploring suicidal feelings and existential issues among the patients. Suicidal communication was sometimes not taken seriously. This type of communication was related to death of undetermined intent rather than suicide. These patients may themselves have been less serious in their intent, and jeopardised their lives with a fatal outcome. Worthy of note is that, overall, they also had higher levels of alcohol in their blood samples at autopsy. Heavy drinking leads to loss of inhibitions and risk-taking, which may trigger self-inflicted death despite less serious intent. It has been shown that those who die by undetermined intent more frequently have alcohol in the blood at autopsy, 62% versus 35% [36], as compared to 73% versus 58% in the present sample, a non-significant difference. Only people with alcohol use disorder were included in the present study, which may explain the higher rates in the suicide group.

The present findings support the hypothesis of the triggering effect of alcohol on suicide and death of undetermined intent [25,26], especially the latter.

Suicide attempt was related to completed suicide, but no relation could be shown with death by undetermined intent. This is in agreement with a recent study [10], which showed a correlation between hospitalisation for self-harm and later suicide, but not undetermined death, in the female group. Other investigators have found suicidal threats (34%) and previous suicide attempts (31%) in cases of undetermined intent, but the sample only included 31% with an alcohol problem [37]. The present findings from the case records are compatible with the results from the staff interviews and autopsy findings of higher blood levels. It indicates a less serious intent in undetermined cases, though self-inflicted death may be triggered by alcohol.

The present study supports the view of a continuum from more ambivalent suicidality in the case of death of undetermined intent to less ambivalent suicidality in the case of suicide, as proposed by other authors in a multicentre study of a general population of self-inflicted deaths [38]. Alcohol use seems to trigger more serious suicidal behaviour. More knowledge is needed about suicidal behaviour as a predictor of death by undetermined intent, and a more thorough clinical investigation of suicidal intent.

The staff knew that the patient was dead, which may have impacted their judgement. The impact of the knowledge is not known. Furthermore, anyone using a rating scale will probably undertake preventative measures, which was not possible in the present study, reducing that confounder.

Another limitation (also often inherent in rating scales) is that the staff did not know the life events that may occur and trigger an accomplished suicide. The impact of traits (such as disease, personality) and states (such as life events) have been discussed in the context of suicide [33]. In most cases only traits or resilience could be judged.

Some people were not remembered, possibly because they had not been to the clinic shortly before death. There were at least similar rates for those who had been in contact within a year and after more than a year, so the effect of recall bias due to time lapse since last contact does not appear to affect the results. However, there is some recall bias due to closer relationships with the staff or more serious illness, which we cannot control for. No scales were used in the present study, so the comparison between staff judgement was made against findings in literature.

The major strength in the present study was the pseudo-prospective design and the use of multiple sources of data, clinically from interviews with staff and case records, as well as autopsy findings.

## 5. Conclusions

The implications of the present study are that clinical predictions by people who know the patient are good without any systematic inquiry (83%–93%) in the case of deciding who is not going to commit suicide. This reflects their intuition about the patient’s resilience. However, the prediction of suicide was poor, less than chance (39%–45%). Suicide scales have also appeared to be inadequate. We propose more active clinical inquiry of suicidal tendencies, especially as people tend to be very private about their suicidal feelings, and also more active follow-up.

Suicidal ideation should be taken more seriously among people with substance use disorders, including those regarded as ‘just a threat’ or ‘only when drunk’. Suicidal ideation of all levels may be predictive of death of undetermined intent, and alcohol appears to trigger fatal suicidal behaviour. Vigorous treatment of alcohol use disorders is also urgent in the ambition to prevent suicide and death by undetermined intent.

## Figures and Tables

**Table 1:** Length of time since last contact with the clinic before death, total sample and patients remembered by staff (%).

Time	All Patients	Patients Remembered
<3 months	98	64 (65)
<1 year	86	43 (50)
<5 years	106	37 (35)
<10 years	44	11 (25)
>10 years	51	2 (4)
Total	385	157 (41)

In three cases the last date of contact was not known.

**Table 2:** Time since last contact with the clinic by manner of death.

Manner of Death	<3 Months	1 Year	5 Years	5–10 Years	>10 Years
Suicide	12 (27)	13 (29)	5 (11)	8 (18)	6 (13)
Undetermined	27 (30)	20 (22)	21 (23)	16 (18)	7 (8)
Other	59 (23)	53 (21)	80 (32)	20 (8)	42 (17)
Total	98 (25)	86 (22)	106 (28)	44 (11)	51 (13)

In three cases the last date of contact was not known.

**Table 3:** Suicidal outcome by manner of death according to staff’s judgement.

Manner of Death	No	Intent Not Considered Serious	Yes	Total
Suicide	11	0	9	20
Undetermined death	30	8 *	9	47
Other	84	4	2	90
Total	125	12	20	157

* intent not considered serious versus suicidal outcome; undetermined death versus suicide, *p* = 0.023.

**Table 4:** Suicidal ideation and suicide attempt by manner of death according to case record evaluation in the long-term course.

Manner of Death	Suicidal Ideation	Suicide Attempt	Total
Suicide	16 (36%)	15 (33%)	45
Undetermined death	24 (26%)	17 (18%)	91
Other manners of death	30 (12%)	42 (17%)	252
Total	70 (18%)	74 (19%)	388
